# Promoting mental health in small-medium enterprises: An evaluation of the "Business in Mind" program

**DOI:** 10.1186/1471-2458-9-239

**Published:** 2009-07-15

**Authors:** Angela Martin, Kristy Sanderson, Jenn Scott, Paula Brough

**Affiliations:** 1 School of Management, University of Tasmania, Hobart, Australia; 2 Menzies Research Institute, University of Tasmania, Hobart, Australia; 3 School of Psychology, University of Tasmania, Hobart, Australia; 4 School of Psychology, Griffith University, Brisbane, Australia

## Abstract

**Background:**

Workplace mental health promotion (WMHP) aims to prevent and effectively manage the social and economic costs of common mental illnesses such as depression. The mental health of managers and employees within small-medium enterprises (SMEs) is a neglected sector in occupational health research and practice, despite the fact that this sector is the most common work setting in most economies. The availability and propensity of SME staff to attend face-to-face training/therapy or workshop style interventions often seen in corporate or public sector work settings is a widely recognised problem. The 'Business in Mind' program employs a DVD mode of delivery that is convenient for SME managers, particularly those operating in regional and remote areas where internet delivery may not be optimal. The objective of the intervention program is to improve the mental health of SME managers, and examine whether employees of managers' whose mental health improves, report positive change in their psychosocial work environment. The mechanisms via which we aim to improve managers' mental health are through the development of their psychological capital (a higher order construct comprised of hope, self efficacy, resilience and optimism) and their skills and capacities for coping with work stress.

**Methods/Design:**

The effectiveness of two versions of the program (self administered and telephone facilitated) will be assessed using a randomised trial with an active control condition (psychoeducation only). We aim to recruit a minimum of 249 managers and a sample of their employees. This design allows for 83 managers per group, as power analyses showed that this number would allow for attrition of 20% and still enable detection of an effect size of 0.5. The intervention will be implemented over a three month period and postal surveys will assess managers and employees in each group at baseline, intervention completion, and at 6 month follow up. The intervention groups (managers only) will also be assessed at 12 and 24 month follow-up to examine maintenance of effects. Primary outcomes are managers' levels of psychological capital (hope, resilience, self-efficacy and optimism), coping strategies, anxiety and depression symptoms, self-reported health, job satisfaction and job tension. Secondary outcomes are participating managers subordinates' perceptions of manager support, relational justice, emotional climate and job tension. In order to provide an economic evaluation of the intervention, both employees and manager rates of absenteeism and presenteeism will also be assessed.

**Discussion:**

The intervention being trialled is expected to improve both primary and secondary outcomes. If proven efficacious, the intervention could be disseminated to reach a much larger proportion of the business community.

**Trial registration:**

Current controlled trials ISRCTN 62853520

## Background

The World Health Organization (WHO) predicts that depression will be ranked as the second largest cause of burden of disease by 2020. The disorders grouped under the generic category 'depression' are the most prevalent psychiatric conditions, with individual lifetime risk estimates ranging between 20–55% [1] In addition to its considerable social impact, depression has significant economic costs related to work performance [2-4], workplace safety, absenteeism, and early retirement [5]. Accordingly, workplace mental health promotion programs are becoming more widespread, but still lack accumulated empirical evidence of their effectiveness.

Research indicates that the early identification of depression symptoms and the provision of encouragement to seek treatment are cost-effective methods for increasing employee well-being [6]. However, it is often argued that we need to go further, by examining those aspects of work and work environments which promote or detract from mental health [7]. There is now a strong body of longitudinal evidence that causally implicates aspects of the work environment in the development of health conditions such as depression (see recent reviews [8,2,9]). Managerial style has been identified as a core concern in occupational health research. For example, managers' lack of support and inconsiderate or hostile behaviour contributes directly to employee depression [10]. The relationship between employee reports of unfair treatment from supervisors and their subsequent increased risk of developing psychiatric problems are observed even when individual dispositions and demographic characteristics are controlled for [11].

The ability of managers to provide social support and create a positive work environment is negatively impacted when their own mental health is compromised. Managers experiencing symptoms of depression are more prone to aggressive behaviour and other forms of abusive supervision [12] and are less likely to be inclined to provide support to employees [13]. This is a significant problem as depression is currently at above average levels among managers. Prevalence rates for DSM-IV depression diagnoses in the Australian National Mental Health Survey were higher among managers than any other occupational group [14]. Another study in the United Kingdom showed that a third of managers undergoing organizational changes reported mental health levels comparable to, or worse than, psychiatric outpatients [10]. While a small proportion of managers may formally meet the diagnostic criteria for depression, a larger number will experience sub-threshold levels of depression symptoms and high levels of occupational stress which affect their behaviour towards employees. Even if managers are not depressed, their transient negative mood states, such as anger and sadness, affect employees through a process theorized as 'emotional contagion' [15]. Accordingly, researchers have called for more research on the impact of leader emotions and behaviour on employee well-being [16].

Studies of employee psychological distress have typically classified depression as an outcome of the work environment (e.g., [12]). In addition to manager's mental health being a key outcome, in the current research the mental health of the manager is also classified as an *independent *variable that is associated with employee well-being. Thus, in addition to directly improving the psychological health of managers, our intervention is also designed to enhance the psychosocial work environment, thereby reducing employees' risk of experiencing occupational stress and depression.

### Focus on SMEs

Recent Australian research indicated that while 43% of managers agreed that depression was a topic suitable for discussion in the workplace, only 25% had received any training from their organization on mental health issues, and only 33% had a clear policy on mental health [13]. These problems were particularly pronounced in SMEs. Occupational epidemiologists have suggested that SMEs need "special attention because their knowledge, competence and financial resources to carry out interventions are limited" ([17], p. 95). Strategies that are routinely employed by larger organizations such as Employee Assistance Programs, mental health literacy workshops, or stress management training are difficult to implement and are infrequently adopted by SMEs. Smaller organizations also provide an excellent setting for the study of emotional contagion as SME employees are often more proximal to the emotions of their managers. Hence, the leader's influence on the work group's affective climate may be a significant determinant of employee well-being within SMEs. In an era of constrained expenditure on healthcare services, this is anticipated to be a cost-effective method of mental health promotion. In addition, economic evaluation of workplace mental health promotion is rare, and almost non-existent for programs delivered in SMEs [18]. This research will also directly evaluate the cost-effectiveness of the intervention to SMEs using measures of absenteeism and presenteeism designed for this sector. Clearly, the SME sector needs urgent attention in relation to occupational health research and practice [19].

### Aims of the research

**Figure 1:**
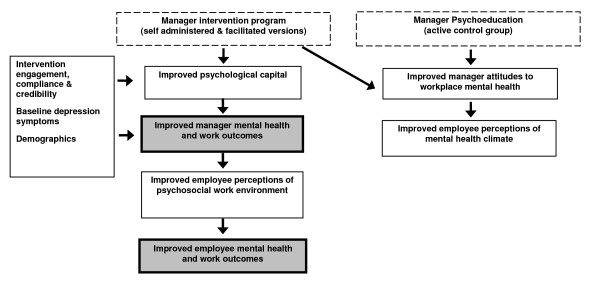
**Proposed Theoretical Model**.

Our study aims to describe the efficacy of a mental health promotion intervention for enhancing the mental health of SME managers and investigate the processes by which the psychological state of managers affects their subordinate's well-being via the workplace psychosocial environment. In summary, our research will test the hypothesized theoretical model shown in Figure 1. In addition, we will calculate the cost-effectiveness of this intervention in order to guide policy and decision-making by our research partners, business stakeholders and government bodies.

## Methods/Design

### Study Design

**Figure 2:**
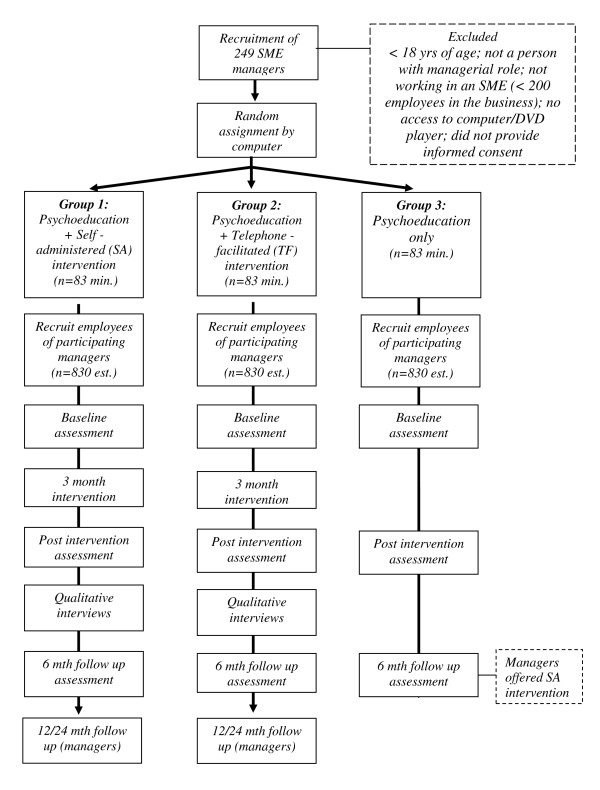
**Study Design Flowchart**.

The research design employed a three arm randomized controlled trial including 249 managers (83/83/83 – see power calculations below). The trial is also evaluated using a sample of employees of each of these managers (estimated to be approximately 2490 employees). Measurements are scheduled at baseline, directly after completion of the intervention, and at 6 months follow up. After this time the managers in the control group will be offered the intervention on a self administered basis (see intervention delivery below). Longer-term follow up measures at 12 and 24 months post intervention will examine changes in managers' risk for depression and the potential for the intervention to protect or 'inoculate' them during significant work or life challenges. Figure 2 presents the flow chart representing the study design.

### Recruitment of participants

We aim to recruit a minimum of 249 managers within SMEs throughout one state of Australia (Tasmania). Managers will primarily be drawn from the networks of three partner organizations collaborating with this research (*beyondblue *– the Australian national depression initiative, Workcover – a statutory occupational health and safety body and the Chamber of Commerce and Industry – a local business association). A mailing list of approximately 1250 managers of SMEs has been compiled from these networks. Cross-sectional self-report surveys of managers obtain an average response rate of 30% [20]. However, given the longitudinal and intervention requirements of this research it is conservatively estimated that 20% of the 1250 managers invited will participate (N = 250 at baseline). Adjusting for attrition we anticipate a sample size of N = 200 at post intervention assessment. Employing Cohen's (1992) formula, a 3 group ANOVA with an effect size of 0.5 would provide a power of 0.8 (*a *= 0.05) with 156 participants (52 per group). We based our anticipated effect size of 0.5 on two meta-analyses [21,22].

Information about the study will also be provided in the media and via local small business associations and potential participants will be invited to contact the research team for more information about the study. To be included, participants must have a managerial role within a business employing less than 200 employees, be over 18 years of age and have access to a telephone and computer/DVD player.

We also aim to recruit an estimated 2490 employees representing workers in SMEs. Participating managers will be asked to facilitate the distribution of research invitations for their employees. Recruitment of employees will be undertaken by the researchers, hence, the decision to participate in the research will be made confidentially by the employees (managers will not know if their employees are participating). All employees of participating managers will be invited to participate. We have based our estimate of the employee sample size on approximately 10 employees per manager agreeing to participate. Small businesses generally employ less than 20 employees and medium sized businesses generally employ less than 200. Whilst it is difficult to estimate, statistics within the region in which we are recruiting indicate that the largest proportion of businesses represented is likely to be categorised as small. As staff turnover in small businesses is expected to be reasonably high, the follow up will be limited to 6 months post-intervention. All participants will be offered the chance to enter a prize draw as an incentive for continued participation.

### Treatment allocation

Randomization by computer will assign managers (and their associated subordinates) to either the experimental or control conditions. No blocking or stratification procedures will be employed as all willing participants who meet the basic inclusion criteria will be eligible for assignment after informed consent is received. Those currently receiving treatment for a mental health disorder will be identified and examined in the final analyses.

## Description of intervention

### Intervention content

The intervention consists of the '*Business in Mind*' DVD program (60 minutes) and accompanying guidebook. The intervention involves skills development using a Cognitive Behaviour Therapy framework and contains four modules. The first module aims to develop participants' understanding of stress and coping processes, introducing the relationships among thoughts, feelings and behaviours. The second module is designed to enhance participants' level of 'psychological capital' (PsyCAP). PsyCAP is a construct drawn from the positive psychology movement and is characterized by the indicators of self efficacy, hope, resilience and optimism. PsyCAP interventions utilize processes of reflection, goal design, pathway generation, overcoming obstacles, role modeling, and the facilitation of preparedness and mastery [23-25]. Module three is focused on overcoming barriers to living a healthy lifestyle and covers topics such as physical activity, nutrition, substance abuse and creating an effective work-life balance. The final module focuses on assisting managers to create a positive work environment and overcome interpersonal stressors by developing their emotional intelligence and communication skills.

The DVD shows real managers sharing their work experiences and demonstrating their skills. The guidebook contains structured tasks and handouts that help participants apply the ideas presented in the DVD to their own situations. An associated password protected website also links participants with additional resources to help them further their personal development. Participants will be given a three month timeline to complete the program.

### Intervention delivery

As stated earlier, face-to-face delivery is not always an optimal method for disseminating evidenced based psychological interventions in SMEs. CBT interventions in organizational settings are typically delivered to small groups of people which are difficult for SME employees to access. Men are particularly hard to reach in this manner with high rates of refusal by men (50–70%) to attend groups administered by mental health services. Face-to-face interventions are also costly to provide; typically involving high salary costs for the specialized delivery. In addition, people living in remote or rural areas are also disadvantaged as services providing specialized programs often do not include remote areas [26]. Thus, there is an urgent need to identify methods to improve the transportability of occupational health programs [27]. As cost-effective interventions are obviously important to increasing service delivery in workplace mental health promotion, a self-administered treatment (SAT) or self-help modality will be developed and tested in this research. SAT versions of CBT usually involve a combination of video material, self-guided workbooks, and telephone support. SATs can reach large numbers of people, are more acceptable to those who would prefer to address emotional issues in the privacy of their own homes and have demonstrated efficacy in reducing levels of psychosocial distress and health-related risk factors in both clinical and non-clinical populations [27,28].

However, there are a number of important empirical questions that need to be answered to facilitate the optimal delivery of self-help programs in the workplace. First, obtaining high levels of employee engagement with workplace mental health programs has been difficult, particularly among men due to mental health stigma. Our research offers an SAT modality in order to overcome this problem but also includes an empirical evaluation of the predictors of treatment credibility, satisfaction and compliance.

Second, contradictory evidence exists concerning the efficacy of a telephone facilitator for boosting the potential treatment power of an SAT [29]. Whilst it might be expected that a telephone facilitated intervention group will show significantly greater impact on outcomes; an effect mediated by greater intervention engagement and adherence; this component increases the cost of delivery substantially and needs to demonstrate a strong economic case to be funded in future trials. This research will formally evaluate the return on investment for two intervention formats (with and without telephone support).

Finally, whether self-help programs should target those with existing problems or symptoms, or else be delivered universally is also uncertain. A recent meta-analysis of self-help programs for depression [30] found that effect sizes were larger in targeted or 'indicated' programs that focus on people with existing depressive symptoms. In contrast, other researchers report that self-help programs are more effective in improving adjustment in individuals with less severe problems [27]. SATs have been found to be particularly useful in the initial or mild stages of mental disorders as they act as a gateway to further therapy for individuals who are hesitant about receiving treatment [31]. This research will investigate the specific mechanisms of intervention effects in order to answer these questions [32].

### Self-administered intervention group

This group will receive a package containing the '*Business in Mind*' DVD program (60 minutes) and accompanying guidebook with instructions about how to progress through the program activities.

### Telephone-facilitated intervention group

In addition to the '*Business in Mind*' program materials, participants in the facilitated condition will also receive six, thirty-minute telephone calls, over a three month period. The telephone calls aim to review tasks and content presented in the DVD, and to address any concerns or difficulties participants encounter engaging with and carrying out the related activities. The process will be guided by a protocol which prompts recall, reviews guidebook tasks, and provides encouragement and assistance. The first call will occur in week one in order to provide an overview of the program, develop some rapport between the participant and facilitator and prompt initial engagement with the intervention materials. Calls two to five occur on a fortnightly basis and are targeted at modules 1–4 of the DVD. In the final telephone call, managers are encouraged to explore ways of generalizing their skills and continuing their personal development.

### Control group

An active control condition consisting of minimal psychoeducation related to depression and the workplace will be employed. The content of this 15 minute DVD and resource folder will be adapted from material delivered in *beyondblue's *workplace training program. This material provides information that helps managers to identify common symptoms of depression in themselves or those under their supervision, and promotes the importance of help-seeking and early intervention. As an active control condition, the content is instructional in relation to mental health literacy and is presented in multimedia format, but does not include any therapeutic/psychological development content.

### Outcome Assessment

A number of measures will be used to assess intervention processes and outcomes.

*Mental health *(employees/managers): Mental health symptoms will be assessed with the K-10 depression/anxiety screening instrument [33].

*Work outcomes *(employees/managers): This will include job satisfaction [34], job Tension [35] and self-reported lost productivity from absenteeism and presenteeism [4].

*Direct effect of intervention *(managers only): The primary outcomes for intervention participants are the Psychological Capital Inventory [24] and the Cybernetic Coping Scale [36]. We will also use a life events scale [37] to examine the protective effects of any psychological capital improvement and the SF-12 as a general indicator of self reported health [38].

*Psychosocial work environment *(employees only): Employees will be asked to report their perceptions of team affective climate [39], relational justice [40], and supervisor support [41].

*Control variables *(employees/managers): the Core-Self Evaluation Scale which measures the emotional stability personality trait [42]; participants will also be asked about any medical or psychological treatment begun or discontinued during the period since the last survey.

*Intervention satisfaction *(managers only): Intervention participants will be asked to report intervention engagement, compliance, credibility [26].

*Psychoeducation effectiveness *(employees/managers):Managers will be asked about their understanding of mental health conditions, attitudes toward depressed employees, their confidence in discussing mental health issues, and help-seeking intentions [13]. Employee perceptions of mental health climate will be assessed by asking participants about their level of confidence in communicating with managers about mental health concerns and the presence of mental health information in their workplace [43].

Demographic data and business descriptors will also be collected.

### Qualitative assessment

In order to gather additional information relevant to understanding intervention processes and effects, telephone interviews will be conducted with participants who volunteer for a follow-up interview 2–4 weeks after the post-intervention survey (both employees and managers). These interviews will assist in understanding participants experiences of the intervention and in dissemination of the intervention beyond the trial setting.

### Treatment adherence and integrity

A process evaluation framework will be employed to test the extent to which the interventions are successfully implemented (i.e. dose delivered, dose received, and fidelity; [44]). The telephone facilitator will self-rate adherence to protocols in delivery as well as their counseling competency skills using validated clinical instruments. All telephone sessions will be audio-taped (subject to participant consent) and rated by the third author in supervision sessions. In addition, two independent experts will rate 10% of randomly selected sessions for treatment fidelity and competence.

### Data analysis

The primary data analysis will compare the efficacy of the two intervention treatment conditions in comparison to the active control group, based on differences in manager's mental health, psychological capital, and work outcomes. Evaluation will be based on an intent-to-treat analysis. A generalised estimating equations approach using the SAS statistical package will be employed. This approach enables the inclusion of all available data including participants who are lost to follow-up or who have incomplete data. Secondary data analyses will test for differences across the groups in employee mental health and perception of the psychosocial work environment, with adjustment for clustering if necessary. Reasons for treatment success/failure will be identified also via a process evaluation framework. An economic evaluation will estimate the incremental cost-effectiveness of the two intervention treatment conditions in comparison to the active control, from both a societal and employer perspective to ensure direct relevance to the SME context. Indirect costs will include lost productive time from absenteeism and presenteeism. Economic analysis will be informed by another of our studies currently underway which is estimating the societal and employer costs associated with depression in the Australian workforce [3].

## Discussion

### Strengths and limitations

The intervention we have developed is based on the integration of a number of evidence based approaches for mental health promotion. To the best of our knowledge, this will be the first RCT of a multilevel mental health promotion intervention that specifically targets SME managers and employees with a unique psychological capital-emotional contagion theoretical mechanism. By integrating current knowledge in clinical and organizational psychology with a public health perspective, the study has also incorporated a number of innovative and significant objectives. First, in addition to directly examining the efficacy of our intervention for improving the mental health of managers, we also aim to further identify the processes by which the psychological state of managers affects their subordinate's well-being via the workplace psychosocial environment. Second, by investigating a range of cost indicators such as absenteeism and presenteeism, we will estimate the economic value of this intervention. Thirdly, we aim to investigate the impact of different intervention formats, the specific problems they address and the characteristics of the participants who respond to them and find them helpful. Each of the objectives will directly inform policy and decision-making by our research partners, business stakeholders and government bodies. It should also be noted that the study protocol was approved by the Human Research Ethics Committee (TAS), which is a joint agreement between the University of Tasmania and the Department of Health and Human Services (DHHS). Funding of the trial, obtained from the Australian Research Council, was awarded via a process of competitive, systematic peer review.

A limitation of this trial is that only people with a computer or DVD player can be included. However, it is estimated that in 2007, 73% of Australian households had a computer and 89% had a DVD player . Whilst ensuring high external validity of our sample, the leniency of the inclusion criteria is also a potential limitation of the study. Not excluding participants previously or currently being treated for mental disorder may be a potential confounder of our results. However, measurement of this status at baseline and throughout the study will allow us to take this into account.

Low participation and/or high drop out rates are often a problem in mental health promotion intervention trials. In an attempt to increase response rate and reduce attrition we have ensured our intervention materials are highly relevant to the business environment and a persuasive rationale for the importance of psychological wellbeing of managers and staff to the bottom line is provided in the recruitment materials. We have also included incentives for continued participation which may help in retaining a reasonable proportion of our control group.

### Future implementation

If this intervention proves to be efficacious in improving the mental health of managers and reducing some of the psychosocial risks of employment that their employees are exposed to, further dissemination of the intervention is anticipated. The research partners would assist in the dissemination process and further government funding for national and international trials would be sought.

## Competing interests

The authors declare that they have no competing interests.

## Authors' contributions

AM designed the study and the intervention program and drafted the manuscript. KS contributed to the design of the study and the intervention program, and revised the manuscript. JS contributed to the design of the study and the intervention program, and revised the manuscript. PB contributed to the design of the study. All authors read an approved the final version of the manuscript.

## Pre-publication history

The pre-publication history for this paper can be accessed here:


